# Towards Deciphering the Hidden Mechanisms That Contribute to the Antigenic Activation Process of Human Vγ9Vδ2 T Cells

**DOI:** 10.3389/fimmu.2018.00828

**Published:** 2018-04-20

**Authors:** Lola Boutin, Emmanuel Scotet

**Affiliations:** ^1^CRCINA, INSERM, CNRS, Université d’Angers, Université de Nantes, Nantes, France; ^2^Sanofi R&D, Biologics Research, Centre de Recherche Vitry Alfortville, Paris, France; ^3^LabEx IGO “Immunotherapy, Graft, Oncology”, Nantes, France

**Keywords:** human γδ T cell, T cell receptor, antigenic activation, phosphoantigens, butyrophilin, B30.2

## Abstract

Vγ9Vδ2 T cells represent a major unconventional γδ T cell subset located in the peripheral blood of adults in humans and several non-human primates. Lymphocytes that constitute this transitional subset can sense subtle level changes of intracellular phosphorylated intermediates of the isoprenoid biosynthesis pathway (phosphoantigens, pAg), such as isopentenyl pyrophosphate, during cell stress events. This unique antigenic activation process operates in a rigorous framework that requires the expression of butyrophilin 3A1 (BTN3A1/CD277) molecules, which are type I glycoproteins that belong to the B7 family. Several studies have further shown that pAg specifically bind to the intracellular B30.2 domain of BTN3A1 linked to the antigenic activation of Vγ9Vδ2 T cells. Here, we highlight the recent advances in BTN3A1 dynamics induced upon the binding of pAg and the contribution of the different subunits to this activation process. Recent reports support that conformational modifications of BTN3A1 might represent a key step in the detection of infection or tumorigenesis by Vγ9Vδ2 T cells. A better understanding of this mechanism will help optimize novel immunotherapeutical approaches that target defined functions of this unique γδ T cell subset.

## 1 γδ T Cells Compose a Special Immunological Unit

Discovered in the mid-1980s, gamma delta (γδ) T lymphocytes still puzzle and fascinate by their unconventional features. During thymic ontogeny, γδ T cell subsets originating from common lymphoid precursor cells emerge before αβ T cells to represent the predominant CD3^+^ population at the fetal development. Their relative frequency then decreases after birth, while αβ T cells progressively predominate. Importantly, for yet unclear reasons, the expression of particular TCR Vγ and Vδ regions is associated with preferential tissue locations. Hence, the major human peripheral γδ T cell subset (frequency >80%) in healthy adult expresses a heterodimeric TCR composed of Vγ9and Vδ2 chains, and represents about 5% of total lymphoïd cells (1, 2). By contrast, Vδ1 and Vδ3 subsets are mainly detected in epithelial tissues, liver, spleen, tonsils, lymph nodes, and thymus (3). Interestingly, γδ T cells compose the majority of circulating T lymphocytes in some non-primate species (i.e., cattle, sheep, pigs, and birds), which raises questions about evolutionary processes and the biology of this subset (4).

From a functional point of view, γδ T cells are involved in the control of microbial infections (e.g., bacteria, virus, and parasite), cell transformation, homeostasis, and tissue repair already reviewed in Ref. (5, 6). Their activation during these physio-pathological contexts induces the release of cytotoxic and bacteriostatic molecules, such as perforin, granzymes, granulysin, and defensins, death-inducing receptor, and TNF-related apoptosis-inducing ligand receptor (TRAIL). Activated γδ T cells also regulate immune responses by secreting a large panel of soluble molecules, such as cytokines, that can promote the clearance of either intracellular pathogens (e.g., TNFα, IFNγ), extracellular bacteria, fungi (IL-17) or parasites (e.g., IL-4, IL-5, and IL-13); inflammatory (e.g., TNFα, IFNγ) or anti-inflammatory responses (e.g., TGFβ, IL-10); tissue healing, epithelium repair, and cell survival. Interestingly, complementary studies have shown that activated γδ T cells, through type I IFN sensitizations, also promote dendritic cells (DC) maturation and, therefore, could represent adjuvant cells (7–9). Moreover, some γδ T cell subset, like human Vγ9Vδ2 T cells, can acquire an antigen-presenting cell (APC)-like phenotype and regulate conventional CD4^+^/CD8^+^ αβ T cell responses (10).

In contrast to most conventional αβ T cells which directly recognize antigenic structures composed of proteasome-generated peptides and polymorphic presenting molecules, that are related to the major histocompatibility complex (MHC) family (e.g., MHC class I/II molecules), the antigenic activation of γδ T cells is mostly MHC-independent, which strengthens their therapeutical interest (i.e., lack of alloreactivity) (11). The antigenic activation of γδ T cells is linked with their tissue residency and the Vδ chain expressed (12, 13). Interestingly, several studies have now reported that γδ T lymphocyte subsets can be activated by various native or modified molecules that mainly derive from a *Self* origin, including MHC-like molecules in mice (e.g., T10–T22) and in humans (e.g., MICA/B, CD1c, CD1d, and EPCR) (14–18) and yet unrelated native molecules, such as F_0_–F_1_ ATP synthase, phycoerythrin, and apolipoprotein A-I (19). More recently, Annexin-A2, which is expressed in tumor cell(s) upon oxidative stress, has been shown to be directly recognized by human Vγ8Vδ3 T lymphocytes (20). TLRs, dectins, and NLRs may act as γδ TCR costimulator (21). Of note, in most cases, the γδ TCR-dependent activation is also tightly regulated by a set of various molecules, including TLRs, dectins, and NLRs, killer Ig-like receptors (e.g., KIR2D, KIR3D), C-type lectins (CD94/NKG2A-C, NKG2D), and several costimulatory molecules shared with αβ T cells (e.g., LFA1, CD2, CD27, and CD28) (22). In this review, we focus our analysis on the γδ TCR-dependent activation modalities of the major peripheral Vγ9Vδ2 T cell subset.

## 2 Human Vγ9Vδ2 T Cells are Specifically Activated by Phosphoantigens

In healthy adult primates, the major peripheral γδ T cell subset, which expresses a TCR composed of Vγ9 and Vδ2 chains, does not account for more than 10% of the total peripheral T cell pool. Interestingly, this lymphocyte subset expands upon microbial infections (e.g., *Mycobacterium leprae, Mycobacterium tuberculosis*) (23, 24). *In vitro* assays that rely on the incubation of peripheral lymphoid cells with mycobacterial lysates have evidenced Vγ9Vδ2 T cell expansion mediated by protease-resistant and phosphatase-sensitive components, hereafter called phosphoantigens (pAg) (25). These low molecular weight agonists are constituted of alkyl esters associated with a diphosphate moiety that carries their bioactivity (26–28). Isopentenyl PyroPhosphate (IPP), which was the first natural pAg identified from the mycobacteria *M. smegmatis*, is also synthesized in eukaryotic cells where it is an intermediate metabolite of the isoprenoid mevalonate (MVA) pathway leading to cholesterol synthesis (29). Several natural pAg have been further identified and characterized from vertebrates (e.g., DMAPP, dimethylallyl pyrophosphate) and microbes (e.g., HDMAPP/HMBPP, 4-hydroxy-3-dimethylallyl pyrophosphate). These microbial metabolites, produced from the DOXP/MEP (1-deoxy-d-xylulose-5-phosphate/2-C-methyl-d-erythritol-4-phosphate) pathway (30–32), are much more efficient to activate Vγ9Vδ2 T cells than MVA-derived IPP. This property could explain the strong reactivity displayed by Vγ9Vδ2 T cells in infectious contexts (33). Dysregulation of the eukaryotic MVA pathway, which leads to an intracellular accumulation of IPP, has been reported in various types of tumor cells (34). For example, the over-expression of HMG-CoA reductase, in non-Hodgkin B cell lymphoma cell-line Daudi or breast adenocarcinoma cells, induces their spontaneous recognition by γ9Vδ2 T cells (35). Accordingly, pharmacological MVA pathway inhibitors that target upstream (e.g., statins) or downstream (e.g., aminobisphosphonates) IPP synthesis, respectively, suppress or trigger pAg-induced Vγ9Vδ2 T cell activation (36).

Primate Vγ9Vδ2 T cells can specifically sense weak modifications of the expression of *Self* molecules, such as pAg, in a contact- and TCR-dependent manner. However, the mechanisms and pathways involved in this peculiar antigenic activation process remain ill-defined. Despite several attempts, direct interactions between pAg and Vγ9Vδ2 TCR have never been clearly evidenced. While the contribution of additional molecules to this species-specific process has been suggested by various complementary studies [e.g., implication of TCR CDRs (37)], this had not been shown until the groundbreaking evidence that butyrophilins could represent a first group of key molecules.

## 3 The Butyrophilin BTN3A1 Orchestrates Vγ9Vδ2 T Cell Antigenic Activation Induced by Phosphoantigens

**Figure 1:**
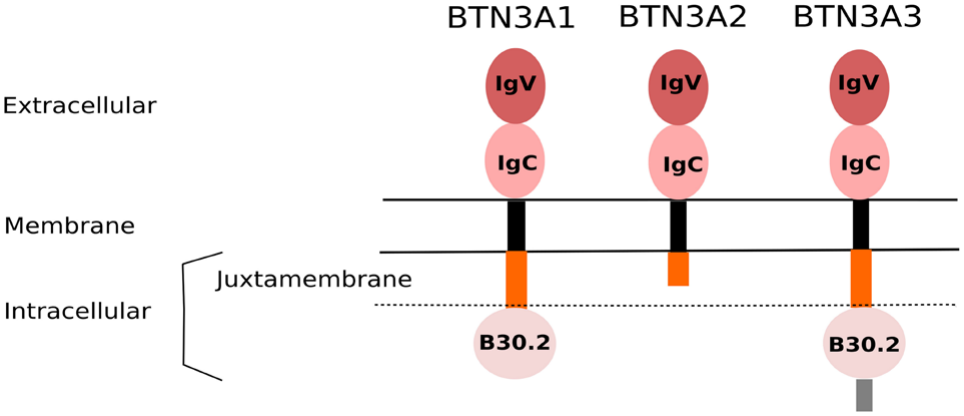
Domain organization of BTN3A isoforms. The BTN3A family proteins share a high structural homology, mainly through their extracellular domain comprising a membrane-proximal IgC and a N-terminal IgV domains. They are linked to a poorly conserved intracellular part *via* a single transmembrane structure (black). The submembrane region (orange) represents the juxtamembrane domain (JTM). BTN3A1 and BTN3A3, but not BTN3A2, contain an intracellular B30.2 domain. The BTN3A3 isoform is further composed of an additional C-terminal extension (gray).

Following the key identification of pAg as potent and specific agonist compounds, the clear evidence that butyrophilin-3A (BTN3A/CD277) molecules also play a mandatory role in the antigenic activation of primate Vγ9Vδ2 T cells was a groundbreaking step to better understand this peculiar and mysterious immunological process (38). Phylogenetically, ubiquitously expressed type I glycoprotein butyrophilin (BTN) molecules share a common ancestor with other members of the B7-CD28 superfamily, which thus suggests that they display immunological functions (39). Indeed, various studies suggest that BTN, as well as BTN-like (BTNL) molecules are involved in some regulatory processes by triggering yet unclear pathways (40). In humans, BTN genes (>10) are located in the telomeric part close to HLA class I region of the chromosome 6p. BTN molecules, which are structurally highly homologous, are divided in three subfamilies (BTN1, BTN2, and BTN3). The BTN3A (CD277) subfamily, then contains three isoforms BTN3A1, -A2, and -A3, belonging to the immunoglobulin (Ig) superfamily and sharing a high structural homology for the extracellular domain composed of an Ig-like IgV and an IgC domain (39) (Figure 1). Of note, the sequences of both B7 and BTN receptors are sufficiently distinct to prevent the latter ones from binding to known costimulatory T ligands, such as CD28 or CTLA-4 (41, 42). While the ectodomain of the three BTN3A isoforms have a very high homology (>95%), only BTN3A1 and BTN3A3 isoforms express an intracellular portion which is composed of a poorly conserved PRY/SPRY B30.2 (hereafter called B30.2) (43). The B30.2 domain, described as a key region for mediating protein–protein interactions, is shared by other BTN family members, as well as various “immunological” proteins, such as TRIM (TRIpartite Motif) and pyrin families (44). The human genome contains four identified *btnl* genes, with the designations of BTNL2, -3, -8, and -9. *btnl2*, the best characterized family member, is clustered with the *btn* genes on chromosome 6, but near to human MHC class II region, whereas the least explored family members *btnl3, btnl8*, and *btnl9* are localized on chromosome 5 (45). Interestingly, BTNL molecules share an homology with the murine Skint family molecules and more particulary with Skint1, which drives the intrathymic differentiation of murine Vγ5Vδ1 T cells (46). The biological function of BTN3A1 molecules is elusive. A recent report shows that BTN3A1 is a positive regulator of the nucleic acid-mediated type I IFN signaling pathway. Upon nucleic acid stimulation, BTN3A1 moves along microtubules toward the perinuclear region, where it directs the interaction of TBK1 with IRF3, thereby facilitating the phosphorylation of IRF3. This process is controlled by microtubule-associated protein 4 (MAP4) (47). Our group described the specific and mandatory contribution of BTN3A1, expressed at the membrane of cellular targets, to the pAg-induced reactivity of primate Vγ9Vδ2 T cells (38). BTN3A1 is ubiquitously expressed in primates, which seems associated with the presence of pAg-reactive γδ T cells in these species (48, 49). Accordingly, BTN3A1 orthologs are not expressed in the rodent lineage that lacks Vγ9Vδ2 T cell counterparts specific for pAg. The emergence of Vγ9Vδ2 TCR and BTN3 molecules with eutherian placental mammals has been reported, suggesting a strong co-evolutionary link (50).

The combined mode of action of BTN3A1 and pAg molecules for triggering a strong and specific antigenic activation of primate Vγ9Vδ2 T cells remains unclear and controversial. BTN3A1 has been first proposed as a “classical” antigen-presenting molecule for pyrophosphate compounds. In this model, pAg would bind a shallow groove within the distal IgV extracellular domain, which induces the formation of stable complexes that then directly interact and stimulate the Vγ9Vδ2 TCR, similarly to the peptide-MHC molecules and αβ TCR system (51). This model, which implies cognate physical interactions between a conserved portion of the IgV domain of BTN3A1, pAg, and the Vγ9Vδ2 TCR, diverges from the data published in several studies that clearly show the key specific requirement for the BTN3A1 isoform in the pAg-mediated activation of Vγ9Vδ2 T cells. In line with these observations, our model proposes that the intracellular B30.2 domain of the BTN3A1 isoform, but not BTN3A3, drives Vγ9Vδ2 T cell antigenic activation through a direct binding of pAg to a charged groove. This model, based on the intracellular sensing of pAg by BTN3A1 molecules, has been supported by several complementary observations. Depletion, domain swapping, and mutation experiments indicate that BTN3A1 lacking its intracellular domain B30.2, or expressing a BTN3A3 B30.2 domain, or at least mutated on some of its critical pAg-binding residues, fail to trigger an efficient pAg-induced Vγ9Vδ2 T cell stimulation. Conversely, chimeric BTN3A3 molecules that expressed the B30.2 domain of BTN3A1 efficiently trigger a pAg-mediated activation (52, 53). Together, these results support the mandatory role played by the intracellular BTN3A1 B30.2 domain in the pAg-binding and sensing by Vγ9Vδ2 T cells.

## 4 B30.2, The Lock/Key System of Intracellular Phosphoantigen Sensing

**Figure 2:**
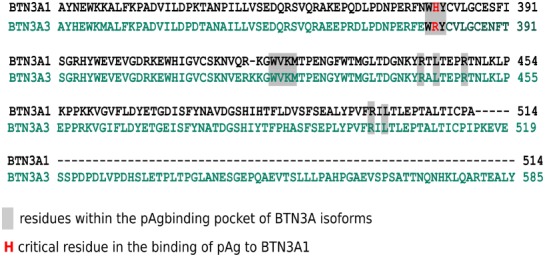
Alignment of the intracellular B30.2 domain sequences of BTN3A1 and BTN3A3 isoforms. Amino acid sequence alignment of the B30.2 domains of BTN3A1 (top line) and BTN3A3 (bottom line). Dashes indicate absent residues in BTN3A1. Amino acids are shown in the single letter designations and numbered according full length nomenclature. Gray boxes indicate residues which constitute the pAg-binding positively charged pocket. Red font highlights the single amino acid difference between pAg-binding pocket, in position 381, H in BTN3A1, and R in BTN3A3 isoforms adapted from Ref. (53).

While the B30.2 domains of BTN3A1 and BTN3A3 display a strong homology (approximately 87% amino acid identity), the domain of BTN3A3 fails to efficiently bind pAg and to trigger a significant antigenic activation of Vγ9Vδ2 T cells. The crystal structure of the B30.2 domain of BTN3A1 gave key information about pAg binding site. Importantly, specificity of the BTN3A1 B30.2 domain is a highly positive charged pocket which is constituted by basic residues, including arginines (R^442^, R^448^, and R^499^), histidines (H^381^ and H^408^), and lysine (K^423^). Positively charged B30.2 domain represents an ideal pocket candidate for binding negatively charged pAg. Accordingly, the mutation from basic to (negatively charged) acidic residues completely abrogates pAg-binding and Vγ9Vδ2 T cell activation (53, 54). However, these results did not entirely explain the differences between the capacity of BTN3A1 and BTN3A3 to bind pAg. Close examination of the amino acid differences between these isoforms revealed a single amino acid difference in position 381 within the binding pocket: a histidine in BTN3A1 and an arginine in BTN3A3 (Figure 2). Swapping this single amino acid between the domains of each isoform (i.e., mutating the H into R in BNT3A1 and R into H in BTN3A3) transferred both binding and functional abilities to stimulate Vγ9Vδ2 T cells. Affinity differences have been measured between endogenous and exogenous pAg by a technique called Isothermal Titration Calorimetry (ITC): KD ≃1 mM for endogenous and KD ≃1 mM for exogenous pAg (55). These results also confirmed that the functional potency of those compounds in mediating activation of Vγ9Vδ2 T cells despite is not directly proportional to the affinity (56). The endogenous IPP is typically 100,000-fold weaker potency than the exogenous HMBPP (57).

Adams and colleagues have investigated the effects of pAg binding to the intracellular domain B30.2 of BTN3A1 by NMR spectrometry and molecular dynamics simulation. Using a BTN3A1 full-length intracellular domain model, they have shown that pAg binding induces conformational changes of the BTN3A1 B30.2 domain. The Y^382^ residue, close to the positive pocket, has been identified as critical by showing the largest perturbation induced by pAg binding. ABP-treated Y352A mutants are less capable of mediating Vγ9Vδ2 T cell activation than the wild-type B30.2-containing protein (58). These conformational changes could represent the first signals delivered to distinguish activating or non-activating molecules. In fact, the BTN3A1 B30.2 domain is able to bind additional negatively charged small molecules, like malonate, citrate, adenosine-diphosphate (ADP), and nucleotidic pAg (59). Exogenous pAg (e.g., HMBPP/HDMAPP) induce a chemical shift into the B30.2 domain that extended to the binding site. IPP binding, a less affine pAg, induces similar shift perturbations but qualitatively smaller in magnitude. These results confirmed the antigenic potential difference between exogenous and endogenous pAg. In contrast, titration of the B30.2 domain with malonate and citrate revealed only few chemical shifts with a small magnitude. Both of these nonantigenic molecules failed to induce perturbations in residues more distal to the pAg-binding site. Strikingly, the conformational changes induced by ADP occur in a different direction than those with pAg (60). NMR and crystallography studies suggest that a precise conformation of BTN3A1 B30.2 domain is required to induce Vγ9Vδ2 T cell activation.

Studies from Massaia’s group provided further mechanistic inputs about the contribution of BTN3A1 in pAg-induced Vγ9Vδ2 T lymphocyte activation. They showed that ABP-treated dendritic cells (DC) release extracellular IPP that can induce a significant Vγ9Vδ2 T cell proliferation (61). They identified the ATP-binding cassette transporter 1 (ABCA1) as a major complex involved in this extracellular release of IPP by ABP-treated DCs, with the physical cooperation of BTN3A1 and apolipoprotein A-I (ApoA-I) molecules (62). BTN3A1 is physically linked to ABCA1 but not associated with ApoA-I. Gene silencing of BTN3A1 in ABP-treated DCs slightly decreased the amounts of IPP released. This important study highlighted the existence of pAg membrane transporter complexes that are involved in the export of these compounds. Conversely, the ways by which external charged pAg could cross the plasmic membrane to reach intracellular butyrophilins and then induce the reactivity of Vγ9Vδ2 T cells remain unclear and will need to be further defined.

## 5 The Juxtamembrane Domain of BTN3A1, Another Key Player in the Sensing of Phosphoantigens

**Figure 3:**
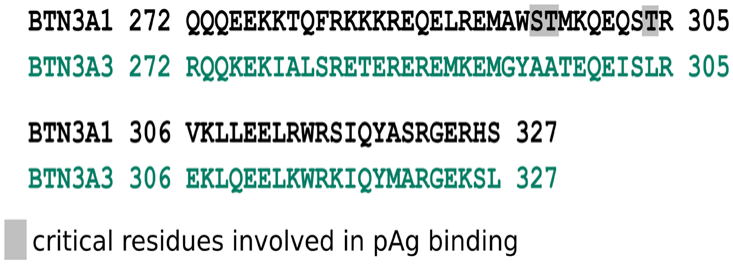
Alignment of sequences encoded by the intracellular juxtamembrane (JTM) domain from BTN3A1 and BTN3A3 isoforms. Amino acids sequence alignment of the JTM domains of BTN3A1 (top line) and BTN3A3 (bottom line). Amino acids are shown in the single letter designation and numbered according to full length nomenclature. Gray boxes show key residues, Ser/Thr^296/297^ and Thr^304^, involved in pAg binding in the JTM region due to the folding of the B30.2 domain in Vγ9Vδ2 T cell antigenic activation adapted from Ref. (64).

The role played by the extracellular and intracellular B30.2 domains of BTN3A1 in the antigenic activation of human Vγ9Vδ2 T cells has been extensively studied. Moreover, the contribution of additional portions of these molecules, such as the juxtamembrane (JTM) domain, has also been carefully analyzed. The JTM domain of many transmembrane receptors, such as growth factor receptors, has been shown to be involved in signaling processes (63). The intracellular JTM region of BTN3A, which is a rather flexible structure, connects the transmembrane domain to the B30.2 one. Our functional activation assays performed with BTN3A1 chimeras swapped for their JTM region support that this intracellular part is a strong regulator of the Vγ9Vδ2 T cell activation. Indeed, BTN3A1 chimeras that express the JTM domains from BTN1A1, BTN2A2, BTNL3, or BTNL9 fail to trigger the antigenic activation of Vγ9Vδ2 T cells. Interestingly, chimeric BTN3A1 molecules expressing the JTM domain of BTN3A3 induce a massive antigenic activation of Vγ9Vδ2 T cells more efficiently than wild-type BTN3A1 (64) (Figure 3). Accordingly, a very recent report further supports these observations by showing that the binding of pAg, such as HMBPP, to the B30.2 domain perturbs residues within the JTM region, suggesting ligand-induced conformational changes. Interestingly, HMBPP could interact with residues within both the B30.2 and the JTM region at different contact points. Furthermore, this report also indicates that both key residues Ser/Thr^296/297^ and Thr^304^ fall within a critical functional BTN3A1 JTM region (65).

The binding of pAg to the intracellular B30.2 domain has been shown to induce conformational changes of the JTM that could have important functional consequences (55). For example, these modifications could either spread to the extracellular domain of BTN3A molecules or alter its membrane topology and dynamics, leading to their recognition by γδ T cells.

## 6 The Holy Grail: Understanding the Cryptic Mechanism of Antigenic Activation of Vγ9Vδ2 T Cells

Despite representing significant advances by suggesting that butyrophilins do not operate as “classical” antigen-presenting partners, such as MHC and MHC-like molecules, these recent observations rather complicated the poor understanding of this peculiar antigenic activation process. Ultimately, γδ T lymphocyte immunologists will need to answer to the challenging question about the mechanism(s) by which the Vγ9Vδ2 TCR exquisitely and specifically sense them, following the increase of pAg levels and their association(s) to butyrophilins, to deliver strong and rapid activation signals. From a fundamental point of view, these future analyses should provide evidences about the intracellular trafficking, the dynamics of these molecules (e.g., intracellular/membrane multimeric complexes), the regulation of this process in normal vs. pathological contexts (e.g., tumor cells). Basically, these results should bring some important information about the still unclear biological functions displayed by these molecules. On a more evolutionary side, these results should help to finally provide an unified overview of the antigenic activation process of human and murine γδ T lymphocyte subsets, some of the latter ones being also regulated by non-BTN3A1 butyrophilin-related molecules (e.g., Skint or BTNL8) (66, 67).

To assist these complex deciphering steps, novel elements have been recently brought, such as the recruitment of BTN3A1 partners and the contribution of other isoforms and conformational changes. As BTN3A1 isoforms have not been shown to directly interact with the Vγ9Vδ2 TCR, various independent studies have been conducted to identify and to characterize extra- and intracellular partner molecules. Consequently, different groups first confirmed that the expression of human BTN3A1 molecules in rodent cells may not be sufficient to simply induce the reactivity of primate Vγ9Vδ2 T cells (68). The transfer of human chromosome 6 in those cells, which triggers this species-specific activation, then suggested that partner molecule(s) are encoded by gene(s) located within this chromosome (69). Two studies recently reported that molecular partners, such as RhoB or periplakin, cognately interact with BTN3A molecules (70). RhoB is a small G protein of the Rho GTPase family that regulates actin reorganization, vesicles transport, and apoptosis in transformed cells following DNA damage. RhoB contributes to various cellular events, including cancer progression through multiple pathways by regulating DNA damage responses, apoptosis, cell cycle progression, migration, and invasion (71). Lipid modifications affect main subcellular localizations (i.e., endosomes, Golgi vesicles, and nucleus) of RhoB and its levels are acutely regulated in response to a variety of stimuli. Using a biolayer interferometry approach, Kuball’s group demonstrated that RhoB binds to the full-length BTN3A1 intracellular domain, while binding was significantly reduced to the B30.2 domain alone. However, the precise contribution of RhoB, which is conserved between humans and rodents and encoded by a gene located in chromosome 2, to the activation of Vγ9Vδ2 T lymphocytes is yet unclear and will require a deeper analysis.

The plakin family member, cytoskeleton adaptor protein periplakin (PPL), whose gene is located in chromosome 16, has also been shown to bind to the BTN3A1 JTM (72). PPL might contribute to the formation of responsive and dynamic structures which could implicate both cytoskeleton components (e.g., intermediate filaments, actin) and pAg. Accordingly, our results from fluorescence recovery after photobleaching (FRAP) experiments have shown an immobilization of BTN3A1 molecules linked to pAg sensitization (53). This suggests that the antigenic activation of Vγ9Vδ2 T lymphocytes is linked to the recruitment and containment of BTN3A1 proteins in selected subcellular domains which are located in the vicinity of the plasma membrane and focal adhesions (LB & ES, unpublished observations). However, PPL knockdown using siRNAs had no clearly interpretable effects on BTN3A1-mediated activation of Vγ9Vδ2 T cells. In this work, the main evidence for a functional contribution of these interactions is a correlation between loss of PPL binding and loss of activation, induced by a deletion of either the VKLLEEL JTM stretch (located in exon 5 of BTN3A1) or only of its di-leucine motif. In so far as PPL does not bind to BTN3A3, while active BTN3A3 carrying the R351H mutation efficiently activates Vγ9Vδ2 T lymphocytes, it seems unlikely that PPL is required for the Vγ9Vδ2 T lymphocyte antigenic activation process.

Initial experiments evidenced that agonist #20.1 BNT3A-specific mAbs bind the IgV ectodomain of BTN3A glycoproteins, which leads to the activation of Vγ9Vδ2 T lymphocytes (38, 73). Among various hypotheses, the possibility of mAb-induced conformational changes, which could mimic pAg-induced modifications, deserves attention. The functional impact of pAg-induced changes of the intracellular B30.2 and JTM domains, that could be then transduced to the extracellular domain and trigger the sensing of these modified *Self* complexes by Vγ9Vδ2 T lymphocytes, has been investigated. Accordingly, independent studies have shown that the conformation of both the B30.2 domain and its upstream JTM region vary upon pAg binding (58, 60). An important study has shown that pAg bind the B30.2 pocket and weakly interact with some residues constituting the JTM region. Based on both length and flexibility characteristics, the authors propose that the B30.2 domain of BTN3A1 is moved toward the JTM region and closer to the membrane upon ligand binding. Such intracellular changes would be sensed by γδ T cells through modifications of either the extracellular domain or interactions with other molecular partners (65). Non-BTN3A proteins composed of an intracellular B30.2 domain have been shown to naturally multimerize and this status is important to fulfill their functions (74, 75).

While first studies proposed that the ectodomain of BTN3A exist at the surface into either V-shaped or head-to-tail conformations (49, 76), recent experiments suggest that only the ectodomain adopts a V-shaped conformation (58). Strikingly, this study indicates that the expression of BTN3A1–BTN3A2 heterodimers in lipid nanodiscs is more stable than BTN3A1 homodimers, which suggest a role for the BTN3A2 isoform (which contains no intracellular B30.2 domain). A growing set of studies from various laboratories confirmed that BTN3A1 molecules are mandatory for pAg-dependent activation of Vγ9Vδ2 T lymphocytes. The contribution of BTN3A2 and BTN3A3 isoforms to this process remained to be understood and was analyzed. Initial functional studies, using global, and likely incomplete, BTN3A-knockdown (shRNA delivered by lentivirus) combined to a forced expression of selected isoforms (transfection), first showed that the expression of BTN3A2 and BTN3A3 isoforms is not sufficient to induce the activation of Vγ9Vδ2 T lymphocytes by pAg. So far, the results failed to demonstrate any inhibitory or activatory role played by non-BTN3A1 isoforms. A work from Hayday’s group proposes that BTN3A1 and BTN3A2 heterodimers would contribute to this process according to this model. The ectodomain and the B30.2 domain of BTN3A1 would represent active entities in Vγ9Vδ2 T lymphocyte stimulation, while BTN3A2 isoforms would rather participate by regulating the appropriate routing (e.g., ER trafficking), kinetics, and/or stability of BTN3A1 (77).

**Figure 4:**
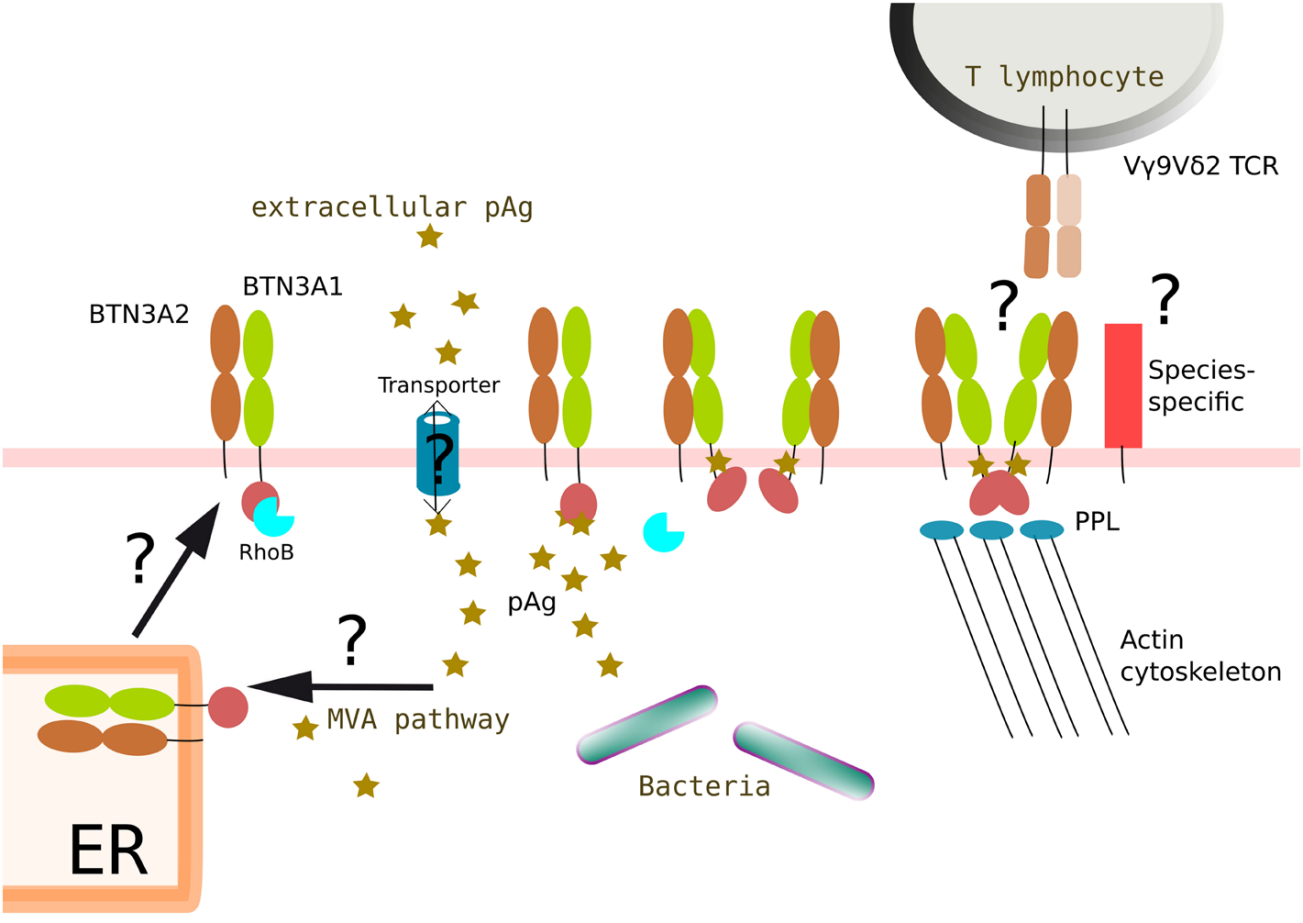
Proposed integrated model of BTN3A1 modifications induced by phosphoantigens and leading to the antigenic activation of Vγ9Vδ2 T lymphocytes. Intracellular accumulation of pAg could originate from a dysregulation of the *Self* mevalonate (MVA) pathway in pathological situations (e.g., cancer, infections) and/or be from exogenous origin (e.g., pathogens). In some situations, negatively charged pAg would need to be routed (e.g., import/export) *via* active processes (e.g., ABC transporters). pAg would then bind to intracellular parts of butyrophilin molecules (e.g., B30.2 ± juxtamembrane domains) at different sub-cellular locations during synthesis and routing steps of the molecules (e.g., ER, cell membrane). The binding of pAg to butyrophilins induces structural modifications that affect the dynamics of the molecules (e.g., membrane diffusion) and the immunological visibility of these molecules. Accordingly, the transition from resting to activatory state of these molecular complexes might also be linked to the nature of the multimerization of BTN3A1 glycoproteins (e.g., homodimers, heterodimers). The contribution of additional partner molecules, some of them being species-specific, regulating actin cytoskeleton modifications (e.g., RhoB, PPL) might also be important. The mechanisms that drive this unique antigenic activation process of human Vγ9Vδ2 T lymphocytes sensing these subtle molecular changes though a specific, contact-, and Vγ9Vδ2 TCR-dependent process remain a major conundrum. The question marks (?) refer to unsolved or yet unclear issues.

Despite these major breakthroughs, the main question remains yet unsolved: how could such conformational changes and heterodimeric associations be specifically sensed by the TCR of Vγ9Vδ2 T lymphocytes. This issue represents major future research tracks in this field (Figure 4). To summarize, complementary research issues can be identified: (i) the subcellular localization for the interactions of pAg with BTN3A molecules; (ii) the role played by additional intra- vs. extracellular partners (e.g., cargo ?); (iii) the characterization of BTN3A antigenic complexes and their dynamics, linked to the antigenic activation status; (iv) the conformation(s)/multimerization of integral BTN3A molecules; and (v) the specific role played by BTN3A2 and BTN3A3 isoforms in these processes.

## Author Contributions

Both authors contributed to the writing process, prepared the manuscript, and approved the final version.

## Conflict of Interest Statement

LB was employed by company Sanofi-Aventis R&D. All other authors declare no competing interests.
